# Probing Cold Supersonic Jets with Optical Frequency Combs

**DOI:** 10.3390/molecules30193863

**Published:** 2025-09-24

**Authors:** Romain Dubroeucq, Quentin Le Mignon, Julien Lecomte, Nicolas Suas-David, Robert Georges, Lucile Rutkowski

**Affiliations:** Université de Rennes, CNRS, IPR (Institut de Physique de Rennes)-UMR 6251, F-35000 Rennes, France

**Keywords:** frequency combs, Fourier transform spectroscopy, supersonic expansion, cold molecules

## Abstract

We report high-resolution, cavity-enhanced direct frequency comb Fourier transform spectroscopy of cold acetylene (C_2_H_2_) molecules in a planar supersonic jet expansion. The experiment is based on a near-infrared frequency comb with a 300 MHz effective repetition rate, matched to a high-finesse enhancement cavity traversing the jet. The rotational and translational cooling of acetylene was achieved via expansion in argon carrier gas through a slit nozzle. By interleaving successive mode-resolved spectra measured at different comb repetition rates, we retrieved full absorption line profiles. Spectroscopic analysis reveals sharp, Doppler-limited transitions corresponding to a jet core rotational temperature below 7 K. Frequency comb and cavity stabilization were achieved through active Pound–Drever–Hall locking and mechanical vibration damping, enabling a spectral precision better than 2 MHz, limited by the vibrations induced by the pumping system. The demonstrated sensitivity reaches a minimum detectable absorption of 7.8 × 10^−7^ cm^−1^ over an 18 m effective path length in the jet core. This work illustrates the potential of cavity-enhanced direct frequency comb spectroscopy for precise spectroscopic characterization of cold supersonic expansions, with implications for studies in molecular dynamics, reaction kinetics, and laboratory astrophysics.

## 1. Introduction

The ability to cool molecules to cryogenic temperatures has revolutionized the field of gas-phase molecular spectroscopy, enabling detailed studies of molecular structure, dynamics, and interactions [1,2]. At low temperatures (and low pressures), the reduced thermal motion suppresses Doppler and collisional broadenings, allowing for high-resolution observation of individual rovibrational transitions [3]. This is particularly beneficial for complex molecules whose room-temperature spectra are highly congested due to the population of many internal states. Consequently, cold molecular spectroscopy has become an essential tool across a wide range of disciplines, including reaction kinetics [4], QED test [5], quantum control [6], intramolecular dynamics [7], molecular clustering [8,9], and laboratory astrophysics [10,11].

To probe the sharp spectral features of cold molecules, high-resolution spectroscopic techniques are required. Conventional approaches, such as Fourier transform infrared (FTIR) spectroscopy [12] and narrowband laser absorption [13], have provided significant insights but often suffer from trade-offs between spectral coverage, sensitivity, resolution, and acquisition time. The emergence of optical frequency combs has overcome many of these limitations. Optical frequency combs generate a broad spectrum of equidistant, narrow-linewidth modes and provide exceptional frequency accuracy and stability, making them ideal light sources for broadband, high-resolution spectroscopy.

Direct frequency comb spectroscopy (DFCS), particularly when combined with optical enhancement cavities, leverages these properties to achieve superior sensitivity and resolution. When applied to cold free jets, DFCS allows for the measurement of individual comb-mode-resolved absorption lines with minimal thermal and pressure broadening. Furthermore, cavity-enhanced DFCS increases the effective absorption length, facilitating trace detection and precise quantification of molecular transitions. Previous work has established the utility of DFCS in buffer-gas-cooled environments and supersonic jets for probing rovibrational transitions under well-controlled conditions. Spaun et al. and Changala et al. employed cavity-enhanced DFCS to study large polyatomic molecules cooled in a cryogenic cell via argon buffer gas, achieving rotationally resolved spectra with exceptional frequency precision [14,15,16]. Specifically, Spaun et al. demonstrated a rotational temperature of approximately 10 K; their setup enabled Doppler-limited resolution and accurate rovibrational line assignments for complex molecules such as nitromethane or naphthalene under collisionally relaxed, low-temperature conditions which enabled resolving the rotational structure of their crowded absorption spectra [14]. A few years later, the same approach applied at longer wavelengths yielded the first rotationally resolved absorption spectrum of the buckminsterfullerene molecule [16].

In this study, we demonstrate near-infrared cavity-enhanced DFCS of acetylene molecules cooled via a planar supersonic expansion in argon. By interleaving spectra recorded at different comb repetition rates, we reconstruct the full high-resolution absorption spectrum of cold acetylene. This approach allows us to extract rotational temperatures below 10 K and resolve contributions from both the isentropic jet core and the surrounding shear layers. We also describe the experimental setup, including mechanical and active stabilization strategies for the optical cavity, and analyze the resulting spectra using Voigt-profile models informed by HITRAN parameters. This work highlights the power of DFCS for precision spectroscopy of cold molecular systems and provides a robust platform for future studies of molecular kinetics and dynamics in non-equilibrium environments.

## 2. Results

We conducted high-resolution absorption spectroscopy of cold acetylene in the near-infrared using a cavity-enhanced frequency comb system. The acetylene sample was cooled via supersonic expansion through a slit nozzle (described in Section 4.2) and probed with a frequency comb featuring an effective repetition rate of 300 MHz. To resolve individual comb modes with high accuracy, we employed the subnominal resolution technique, in which a mechanical Fourier transform spectrometer (FTS) is used to measure the transmitted comb light with a resolution finer than the comb spacing [17].

**Figure 1:**
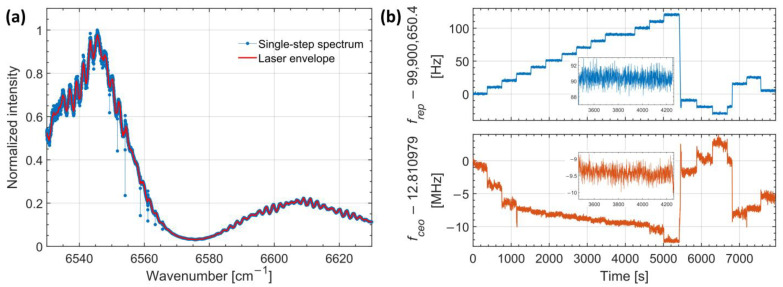
(**a**) Single-step transmission spectrum of cold acetylene recorded with the frequency comb stabilized to a fixed repetition rate. Blue dots show the normalized intensity spectrum after baseline correction; the orange curve represents the underlying laser spectral envelope, highlighting power variations due to the laser gain profile, cavity transmission filtering, and etalon fringes. Distinct absorption features correspond to rovibrational transitions of acetylene, with each line sampling a single comb mode. (**b**) Stability of the frequency comb parameters during the interleaved acquisition. The upper panel shows the comb repetition rate *f*_rep_, stepped in 10 Hz increments across 19 steps to shift the optical comb grid. The lower panel displays the carrier-envelope offset frequency *f*_ceo_ measured concurrently. Insets show zoomed-in views of step 10, centered around 4000 s, illustrating sub-Hz stability for *f*_rep_ and sub-MHz stability for *f*_ceo_, corresponding to an overall optical frequency uncertainty below 2 MHz.

A representative spectrum from a single repetition-rate step is shown in Figure 1a. Sharp absorption features corresponding to acetylene transitions stand out from the broad spectral envelope of the laser. These features confirm that, at each step, only a single comb mode is absorbed per line profile—an effect arising from the narrow linewidths of the cold molecular sample. Notably, variations in the detected intensity across the spectrum are due to the spectral power distribution of the laser source, cavity transmission filtering, and residual etalon fringes. To fully reconstruct each absorption line shape, the measurement must be repeated at multiple comb offset positions, as discussed in the sections that follow.

### 2.1. Frequency Calibration of the Frequency Comb and Stability Performances

Each single-step measurement was conducted with the frequency comb fully stabilized: the repetition rate (*f*_rep_) and carrier-envelope offset frequency (*f*_ceo_) were stabilized and continuously monitored using a frequency counter with a 10 ms integration time (see Section 4). With the comb locked to the enhancement cavity and the cavity length itself actively stabilized, it was possible to precisely vary *f*_rep_ in discrete steps while maintaining comb–cavity resonance.

To recover full absorption line profiles from the cold acetylene sample, we employed the subnominal resolution and interleaving method [17]. The subnominal resolution method takes advantage of the precise frequency spacing of the comb modes to extract spectral information at a resolution finer than the nominal comb spacing. By applying a boxcar apodization function to the symmetric interferogram with an optical path difference interval exactly equal to c/*f*_rep_ (with c, the speed of light), the Fourier transform samples the maxima of the comb modes without introducing cross-talk. Subsequent interleaving, achieved by systematically scanning the comb lines (and corresponding cavity resonances) relative to the molecular transition, enables point-by-point reconstruction of the absorption profile with effective sampling finer than the intrinsic comb spacing. Specifically, *f*_rep_ was incremented in 10 Hz steps, corresponding to 19.6 MHz shifts in the optical domain. A total of 19 steps were performed, sufficient to span the full 300 MHz gap between adjacent transmitted comb modes.

Figure 1b shows the time traces of the stabilized *f*_rep_ (top panel, blue) and *f*_ceo_ (bottom panel, orange) throughout the acquisition. The standard deviation in *f*_rep_ for each step was approximately 0.75 Hz (translating to 1.5 MHz optically), while the typical fluctuation in *f*_ceo_ was 300 kHz. The resulting uncertainty in the absolute optical frequencies of the comb lines remained below 2 MHz (6 × 10^−5^ cm^−1^), ensuring high accuracy in the reconstructed spectra.

### 2.2. Interleaved Normalized Spectrum

For each step in the comb repetition rate, the interferogram was acquired over a total path difference of 1 m and truncated according to the inverse of the corresponding repetition rate (expressed in wavenumber units) and shifted in frequency by the associated carrier-envelope offset, following the subnominal resolution protocol described in Ref. [17]. The resulting spectra were obtained by computing the absolute value of the fast Fourier transform (FFT) of the processed interferograms. To improve the signal-to-noise ratio, 40 interferograms were recorded under identical conditions and averaged at each step.

Prior to spectral interleaving, each individual spectrum was normalized by its baseline, retrieved using a cepstral analysis method [18]. The cepstral method is a signal-processing technique that analyzes a spectrum by taking the logarithm of its magnitude and then performing an inverse Fourier transform, producing the so-called “cepstrum”. This approach helps separate slowly varying spectral envelopes (such as cavity transmission functions) from sharp molecular absorption features, because they appear at different “quefrencies” in the cepstral domain. By filtering and then transforming back, the laser spectral envelope and associated baseline distortions—caused by variations in comb power, cavity filtering, and etalon effects—were effectively removed. Figure 1a illustrates the result for a single repetition-rate step, where the red curve indicates the extracted baseline.

**Figure 2:**
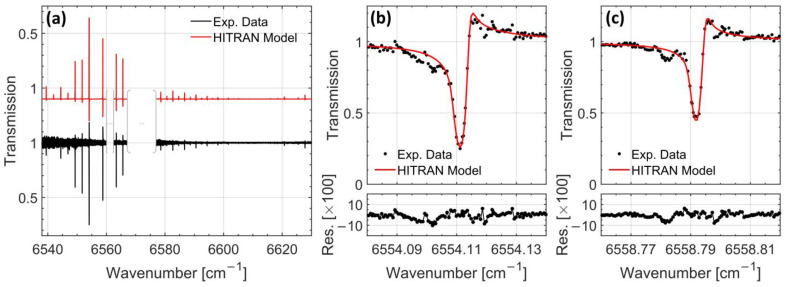
Acetylene spectroscopy. (**a**) Full interleaved and baseline-normalized transmission spectrum of cold acetylene (black) overlaid with a synthetic spectrum (red) generated from HITRAN spectroscopic parameters, fitted Voigt line-shape models, and cavity-enhanced transmission modeling. Spectral regions where the intensity is close to zero are not shown. The HITRAN model has been vertically offset and inverted for clarity. (**b**,**c**) Zoomed-in views of two representative absorption features centered at 6554.11 cm^−1^ and 6558.79 cm^−1^, respectively. Experimental data (black dots) are compared to the fitted HITRAN-based model (red line). Residuals from the fit are plotted below each spectrum, showing minimal deviation and confirming good agreement. Line asymmetries are well captured by the model, which includes the effects of comb–cavity detuning and molecular dispersion.

The complete high-resolution spectrum was reconstructed by interleaving all normalized steps. The resulting composite spectrum is shown in Figure 2a (black trace). Two spectral gaps are visible, corresponding to regions where the laser intensity fell below 5% of its maximum, preventing reliable baseline determination and reducing spectral fidelity in those intervals.

### 2.3. Cold Acetylene Spectroscopy

The strongest absorption features observed between 6545 and 6570 cm^−1^ correspond to the P(3)–P(1) and R(0)–R(3) transitions of the ν_1_ + ν_3_ combination band of acetylene. Additional transitions centered near 6623 cm^−1^, assigned to the P(2), P(1), R(0), and R(1) lines of the ν_1_ + ν_2_ + (ν_4_ + ν_5_)^0^ band, are also clearly resolved. These lines predominantly originate from light interacting with the cold, isentropic core of the supersonic jet. The relative intensities of these features are consistent with a rotational temperature below 10 K. In contrast, weaker absorption lines detected in the 6520–6547 cm^−1^ and 6577–6610 cm^−1^ intervals of the ν_1_ + ν_3_ band are attributed to warmer regions of the jet, specifically the shear layers surrounding the cold core. From the strength and width of these lines, a local rotational temperature of approximately 220 K is inferred. Spectral data near 6575 cm^−1^, where the comb power drops significantly, are excluded from further analysis due to insufficient signal intensity, as indicated by the gaps visible in Figure 2a.

### 2.4. Cavity-Enhanced Absorption Model

To quantitatively interpret the measured transmission spectrum, we modeled the cavity output as arising from two distinct absorbing regions: the cold isentropic core of the supersonic jet and the surrounding warmer shear layers. Many of the observed absorption lines—especially those originating from the jet core—exhibit noticeable asymmetries. These distortions are consistent with slight detunings between the optical frequency comb modes and the cavity resonances, as well as with dispersion effects introduced by molecular absorption, which has been the subject of previous studies [19,20]. Briefly, an intracavity molecular absorber introduces dispersion in the cavity, which translates to a symmetric frequency shift of the cavity modes away from the central frequency of the molecular absorption line. This effect, coupled to a detuning of the comb modes and their respective cavity resonances, yields asymmetric distortions of the absorption line profiles as seen on Figure 2.

The normalized transmitted spectrum was fitted using a cavity transmission model incorporating two Voigt-profile contributions for each line. These contributions represent the cold and warm components of the absorbing medium, and their parameters were constrained using HITRAN spectroscopic data. The two components were assigned independent rotational and translational temperatures, as well as distinct number densities. The cavity finesse was independently determined via Fourier transform cavity ring-down spectroscopy [21], and the spectral variation in the comb–cavity offset was estimated from the asymmetry of the observed line shapes. Both parameters were held fixed during the fitting procedure, which was based on the Python package LMFIT version 1.3.3, implementing the Levenberg–Marquardt method for non-linear least-squares curve-fitting [22]. Free parameters in the fit included the rotational temperatures of the cold jet and shear layers, the Doppler width of the core (used to infer the translational temperature), and the respective molecular number densities. The fitted model is plotted together with the experimental data in Figure 2a. To highlight the good agreement between the experimental data and the model, two acetylene lines are zoomed in on in Figure 2b,c, along with the fit residuum in the lower part of the panel. Nevertheless, interleaving artifacts remain unaccounted for and are observable at the left of the two absorption lines.

## 3. Discussion

A summary of the retrieved and simulated thermodynamic parameters is provided in Table 1. The retrieved rotational temperature of the jet core (6.45 ± 0.02 K) is the lowest temperature reported to date for molecular DFCS, while the extracted core density (5.15 ± 0.02 × 10^13^ molecules/cm^3^) is consistent with estimation based on the sample and carrier gas flow rates and supersonic expansion models. The small discrepancy in translational temperature, with a measured value of 10.22 ± 0.08 K, likely reflects lateral streamlines curvature towards the interior of the flow increasing the observed radial velocity [9]. Indeed, assuming absorption line profiles purely dominated by thermal effects (hence purely gaussian), a radial velocity component of approximately 40 m/s, corresponding to a convergence angle smaller than 5°, would be enough to account for such a broadening of the absorption lines.

Baseline noise levels were evaluated in two spectral regions: 6563.5–6565.5 cm^−1^ and 6612.8–6615.7 cm^−1^, yielding standard deviations of 6.0 × 10^−3^ and 1.4 × 10^−3^, respectively. The lower noise at higher wavenumbers corresponds to the spectral region dominated by absorption from the warmer jet layers and indicates a fourfold improvement in signal-to-noise ratio (SNR). These fluctuations define the minimum detectable absorbance. In the highest-SNR window (≈6613–6614 cm^−1^), the minimum detectable absorbance is αₘᵢₙ × *L*_eff_ = 1.4 × 10^−3^. Using a slit length of *L*_slit_ = 8.1 cm and a measured cavity finesse of *F* ≈ 350, the effective optical path length is *L*_eff_ ≈ 2*F* × *L*_slit_/π ≈ 18 m. This corresponds to a minimum detectable absorption coefficient of αₘᵢₙ ≈ 7.8 × 10^−7^ cm^−1^ and to a minimum detectable acetylene density of 3 *×* 10^11^ molec/cm^3^.

For comparison, Vaernewijck et al. employed a femtosecond-enhanced cavity and FTS to study slit supersonic jets of C_2_H_4_, N_2_O, and C_2_H_2_ in argon [23]. Using a 1 cm slit length, a cavity finesse of approximately 48,960, and an estimated 5 mm absorption path in the jet, they reported a minimum detectable absorption of αₘᵢₙ ≈ 9 × 10^−7^ cm^−1^. While this sensitivity is comparable to our own, their result was achieved with a cavity finesse more than two orders of magnitude higher. This highlights the effectiveness of our approach—particularly the combined active stabilization of both the frequency comb and cavity length—in achieving high signal-to-noise performance with more modest optical finesse.

Our study contributes to the expanding field of high-resolution direct frequency comb spectroscopy (DFCS) of cold molecular systems, with particular relevance to gas-phase molecular physics. Thorpe et al. were the first to demonstrate direct frequency comb spectroscopy in a supersonic expansion, using cavity-enhanced DFCS based on a virtual phased array spectrometer to probe rotationally cooled molecules [24]. However, the divergent nozzle used in this proof-of-concept experiment produced a non-collimated supersonic jet and the retrieved absorption line profiles were significantly distorted by the radial velocity distribution of the acetylene molecules in the jet. They achieved a minimal detectable density of 1.7 × 10^11^ molec/cm^3^ using a 1 cm-wide jet and a 6300-finesse cavity, and they observed a rotational temperature of 9 K along the nozzle axis. In 2022, Agner et al. extended this approach by implementing dual-comb spectroscopy at 8 μm to interrogate a skimmed supersonic jet, achieving rapid, broadband data acquisition over mid-infrared rotational–vibrational transitions of CF_4_ at a rotational temperature close to 10 K [25]. Their configuration enabled probing of the supersonic jet with improved time resolution but at reduced spectral resolution and sensitivity.

In contrast to buffer-gas methods, our system achieves rotational temperatures below 7 K without the need for cryogenics while maintaining high resolution through subnominal-resolution Fourier transform spectroscopy. Importantly, we demonstrate that a relatively modest cavity finesse (~350) suffices to reach absorbance sensitivities on the order of 10^−7^ cm^−1^, owing to the robust stabilization of both the frequency comb and cavity length. This work therefore advances DFCS as a powerful tool for free jet spectroscopy, capable of providing accurate thermodynamic parameters, resolving subtle asymmetries in line shapes due to comb–cavity detuning, and characterizing nonequilibrium flow regions with spatial and spectral precision. It offers a broadly applicable platform for exploring molecular structure, energy transfer, and reaction dynamics in cold, low-density environments.

## 4. Materials and Methods

### 4.1. Optical Frequency Comb Spectrometer

**Figure 3:**
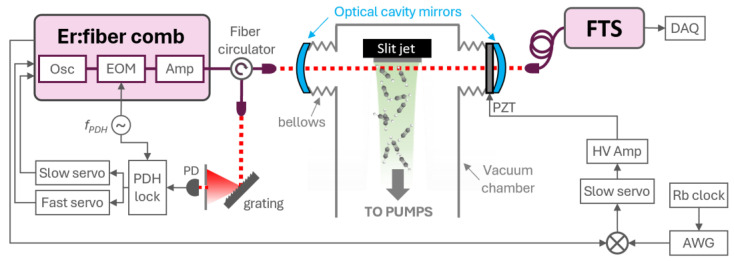
Experimental setup for the comb-jet experiment. Osc, oscillator; EOM, electro-optic modulator; Amp, amplifier; *f*_rep_, repetition rate detection module; PDH, Pound–Drever–Hall electronics; PD, photodiode; PZT, piezo transducer; HV Amp, high-voltage amplifier; FTS, Fourier transform spectrometer; DAQ, data acquisition; AWG, arbitrary waveform generator. See text for details.

The spectrometer used in this work is based on previously established designs [26], adapted for operation in a supersonic expansion environment. The setup consists of a near-infrared optical frequency comb coupled to a Fabry–Perot enhancement cavity, which spans the supersonic chamber. The cavity-transmitted light is analyzed using a fast-scanning Fourier transform spectrometer (FTS), as illustrated in Figure 3.

The frequency comb is generated by an amplified erbium-doped fiber oscillator (ModeHybrid, Mode-Locked Technology, Wroclaw, Poland), operating at a 100 MHz repetition rate with an average output power of 50 mW. To minimize acoustic interference from the vacuum pumps, the comb source is located in a separate room and connected to the supersonic chamber via a 10 m polarization-maintaining optical fiber and circulator. At the chamber entrance, the comb light is launched into free space and mode-matched to the 1 m long Fabry–Perot cavity oriented perpendicular to the jet axis. The cavity comprises two ½-inch curved mirrors (radius of curvature of 1 m) with specified reflectivity between 99% and 99.3% across the 1500–1600 nm range (Layertec, coating 109332, Mellingen, Germany). The cavity free spectral range is intentionally mismatched to the comb repetition rate by a factor of 3/2, resulting in an effective comb mode spacing of 300 MHz in transmission.

The transmitted beam is then recoupled into a second 10 m optical fiber and delivered to the FTS, which is equipped with an analog auto-balanced detector [26]. The detector output, along with a co-propagating reference signal from a HeNe laser used for interferogram calibration, is digitized using a data acquisition (DAQ) system for further processing.

### 4.2. Supersonic Slit-Jet Chamber

The supersonic chamber used in this study has been described in previous works [27]. In the present configuration, a gas mixture consisting of 5% C_2_H_2_ in argon at a backing pressure of 800 Torr was expanded into a vacuum chamber maintained at 0.16 Torr through an adjustable slit nozzle. This nozzle, previously employed for state-resolved spectroscopy of ethylene [28] and detailed in Ref. [29], was configured with an aperture of 8.1 cm in length (along the optical axis) and 30 μm in width. The expansion was performed at a Mach number higher than 10, yielding an almost perfectly unidirectional gas flow perpendicular to the optical axis. The optical probe beam, characterized by a waist radius of 500 µm, intersected the supersonic expansion 2 mm downstream from the nozzle exit. The cold region probed by the optical beam was 8.1 cm long (same as the slit length) and wider than 2 mm on the vertical axis, based on the supersonic jet simulation presented in Ref. [29].

Vacuum conditions were maintained using a multistage pumping system consisting of four parallel Edwards pXH 6000 root blowers (Edwards, Burgess Hill, UK), followed by a secondary Alcatel pump and a primary Busch CLFH 631 pump (Busch Vacuum Solutions, Maulburg, Germany), delivering a total throughput of 21,500 m^3^/h. To suppress mechanical vibrations originating from the pumping system, a 40 cm diameter disc bellows was installed between the pumps and the chamber. This passive damping stage ensured sufficient mechanical isolation, allowing the physical length of the optical cavity to remain stable within 500 nm over one-second time intervals.

### 4.3. Passive Cavity Stabilization

**Figure 4:**
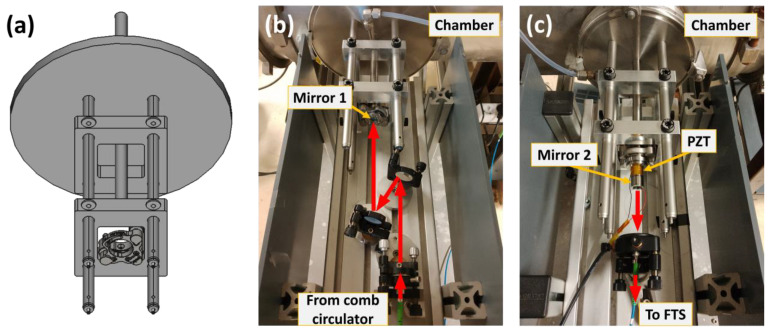
Passive vibration damping: mirror mount cages mounted on the jet cell flanges. (**a**) Mechanical drawing of the cage system. (**b**) Input mirror cage with comb light coupled to free space from fiber and injections mirrors (the red arrows indicate the path of the comb light). (**c**) Output mirror cage (PZT visible, glued to the mount) with collimator and fiber going to the FTS.

To further suppress vibrations induced by the pumping system, a custom mirror mounting system was developed, improving upon previous designs [27] and inspired by the approach detailed in Ref. [30]. A 3D rendering of the mount assembly is shown in Figure 4a. Each optical cavity mirror was supported by a rigid frame constructed from four 25 cm long, ½″-diameter stainless steel posts directly affixed to the chamber flanges. Two square aluminum plates (10 cm × 10 cm × 2 cm) were mounted on the posts: the first to provide structural reinforcement, and the second to hold the mirror housing itself. Both plates were secured in place with spring-loaded thumbscrews to ensure mechanical stability and alignment.

To minimize contamination and maintain optical quality, the structure was aligned with a gas flush tube that delivered nitrogen during operation, preventing the buildup of static gas in front of the mirrors. Each mirror was enclosed in a metallic, vacuum-sealed housing equipped with O-rings and compatible with standard 1″ optical mounts. The housings were connected to the nitrogen flush line via a ½″ flexible disc bellows, which allowed fine alignment without additional gas leakage. Photographs of the input and output mirror assemblies are shown in Figure 4b and Figure 4c, respectively. This mechanically symmetric design ensures that residual vibrations affect both mirrors equally, resulting in relative cavity length stability better than 10 nm over a one-second timescale, even though their absolute positions may fluctuate.

### 4.4. Active Comb-Cavity Stabilization

To maintain continuous and stable transmission through the enhancement cavity, the frequency comb was actively locked to the cavity using the Pound–Drever–Hall (PDH) technique [31,32]. An electro-optic modulator (EOM), integrated within the comb housing, applied a phase modulation at 10 MHz (*f*_PDH_). The reflected light from the cavity was routed through a fibered circulator and demodulated to generate an error signal, which was used to control the comb’s emission parameters. Two proportional-integrator (PI) servos processed this signal to actuate both a piezoelectric (PZT) fiber stretcher and the pump current of the oscillator, with control bandwidths of approximately 30 Hz and 30 kHz, respectively.

This single-point PDH lock ensured precise matching of the comb mode frequencies to the cavity resonance conditions. To implement the subnominal resolution method, the comb repetition rate had to remain absolutely stable throughout each measurement. This was accomplished by referencing *f*_rep_ to a rubidium frequency standard via an arbitrary waveform generator (AWG). The beat note between *f*_rep_ and the AWG reference served as an additional error signal, which was fed to a third PI controller configured as a pure integrator to maintain long-term frequency stability via an annular PZT glued on the output cavity mirror (Mirror 2 in Figure 4c).

## 5. Conclusions

We have demonstrated high-resolution, cavity-enhanced direct frequency comb spectroscopy of cold acetylene in a planar supersonic expansion, achieving rotational temperatures below 7 K. By employing subnominal-resolution Fourier transform detection and interleaving measurements across multiple comb repetition rates, we fully resolved individual rovibrational transitions with sub-MHz accuracy and Doppler-limited resolution.

The spectroscopic data were successfully modeled using Voigt profiles informed by HITRAN parameters, incorporating contributions from both the cold isentropic core and warmer shear layers. Despite the use of a relatively modest cavity finesse (~350), we achieved a minimum detectable absorption coefficient on the order of 10^−7^ cm^−1^—comparable to or better than earlier works employing significantly higher finesse cavities.

This work illustrates the strength of cavity-enhanced DFCS as a powerful tool for studying cold molecules in supersonic jets, providing high sensitivity, spectral resolution, and flow diagnostics within a compact, non-cryogenic setup. The demonstrated approach opens new avenues for precision spectroscopy of transient species, molecular clusters, and reaction intermediates under non-equilibrium conditions relevant to atmospheric chemistry, combustion, and astrophysical environments.

## Figures and Tables

**Table 1:** Thermodynamic conditions in the supersonic jet: temperatures and densities obtained from spectral fitting. The width of the isentropic core was assumed to be equal to the slit nozzle length, while the extent of the surrounding shear layers was set to 1 cm (their effective length is not an important parameter in the spectroscopic model). The experimental parameters were retrieved from Voigt-profile fits to the cavity-enhanced transmission spectrum. Uncertainties (in parentheses) represent one standard deviation in the least significant digits and correspond to the statistical uncertainty from the fitting algorithm.

Jet Region	Rotational Temperature	Translational Temperature	Density
Isentropic core	6.45(2) K	10.22(8) K	5.15(2) × 10^13^ molec/cm^3^
Shear layer	211(2) K	211(2) K	4.57(5) × 10^14^ molec/cm^3^

## Data Availability

The data that support the findings of this study are available from the corresponding author upon reasonable request.
